# Catestatin: Antimicrobial Functions and Potential Therapeutics

**DOI:** 10.3390/pharmaceutics15051550

**Published:** 2023-05-20

**Authors:** Suborno Jati, Sumana Mahata, Soumita Das, Saurabh Chatterjee, Sushil K. Mahata

**Affiliations:** 1 Department of Chemistry and Biochemistry, University of California San Diego, La Jolla, CA 92093, USA; sjati@ucsd.edu; 2 Department of Medicine, University of California San Diego, La Jolla, CA 92093, USA; sumahata@health.ucsd.edu; 3 Department of Biomedical and Nutritional Science, University of Massachusetts Lowell, Lowell, MA 01854, USA; soumita_das@uml.edu; 4 Department of Medicine, University of California Irvine, Irvine, CA 92697, USA; saurabhc@hs.uci.edu; 5 VA San Diego Healthcare System, 3350 La Jolla Village Drive, San Diego, CA 92161, USA

**Keywords:** Chromogranin A, catestatin, gut microbiome, antimicrobial peptide, cell permeable peptide

## Abstract

The rapid increase in drug-resistant and multidrug-resistant infections poses a serious challenge to antimicrobial therapies, and has created a global health crisis. Since antimicrobial peptides (AMPs) have escaped bacterial resistance throughout evolution, AMPs are a category of potential alternatives for antibiotic-resistant “superbugs”. The Chromogranin A (CgA)-derived peptide Catestatin (CST: hCgA_352–372_; bCgA_344–364_) was initially identified in 1997 as an acute nicotinic-cholinergic antagonist. Subsequently, CST was established as a pleiotropic hormone. In 2005, it was reported that N-terminal 15 amino acids of bovine CST (bCST_1–15_ aka cateslytin) exert antibacterial, antifungal, and antiyeast effects without showing any hemolytic effects. In 2017, D-bCST_1–15_ (where L-amino acids were changed to D-amino acids) was shown to exert very effective antimicrobial effects against various bacterial strains. Beyond antimicrobial effects, D-bCST_1–15_ potentiated (additive/synergistic) antibacterial effects of cefotaxime, amoxicillin, and methicillin. Furthermore, D-bCST_1–15_ neither triggered bacterial resistance nor elicited cytokine release. The present review will highlight the antimicrobial effects of CST, bCST_1–15_ (aka cateslytin), D-bCST_1–15_, and human variants of CST (Gly364Ser-CST and Pro370Leu-CST); evolutionary conservation of CST in mammals; and their potential as a therapy for antibiotic-resistant “superbugs”.

## 1. Introduction

Microbial infections in critically ill patients are a global threat. With failing host defense, the use of antibiotics has taken the place for containment of those infections. Nevertheless, the microbes have also evolved with time to develop resistance against those drugs. This arm’s race between antibiotic drugs and pathogens had led to the rise of multi-drug-resistant microbes, called “superbugs”, which emphasizes the urgent need to develop new modes of treatment. Since antimicrobial peptides (AMPs) have evaded bacterial resistance for millions of years of evolution [1], AMPs could be a potential solution for antibiotic resistant “superbugs”. Chromogranin A (CgA), the acidic and secretory proprotein [2,3], is proteolytically cleaved to generate several biologically important peptides, including Catestatin (CST: hCgA_352–372_) [4,5,6,7,8,9,10,11,12]. The 21 amino acid peptide CST (human: S_352_SMKLSFRARAYGFRGPGPQL_372_; bovine: R_344_SMRLSFRARGYGFRGPGLQL_364_) was identified in 1997 as a physiologic brake in catecholamine secretion, which acts by non-competitive inhibition of nicotinic-cholinergic signaling [5,13,14,15,16,17,18]. CST is now established as a pleiotropic peptide [6,19,20]

The non-hemolytic antimicrobial (bacteria, fungus, and yeast) effects of CST (bCST_1–15_ aka cateslytin) were first reported in 2005, where CST was shown to act by penetrating fungal and yeast cell membranes [21]. The study showed that less than 10 µM peptide was required to kill the bacteria. Antibacterial activities were also reported for the two human variants of CST (G_364_S-CST and P_370_L-CST) [22] with minimal inhibitory concentration (MIC) of 1–20 µM [21]. Later, D-bCST_1–15_ (where L-amino acids were changed to D-amino acids: r_344_smrlsfrargygfr_358_) was shown to exert more potent antibacterial (both Gram-positive and Gram-negative bacteria) effects than natural CST [23]. The present review will focus on the antimicrobial effects of CST, with special emphasis on the mechanisms underlying its antibacterial effects, therapeutic potential, and evolutionary conservation.

## 2. Antibacterial Effects of CST

### 2.1. Inhibition of Bacterial Growth by CST

**Figure 1:**
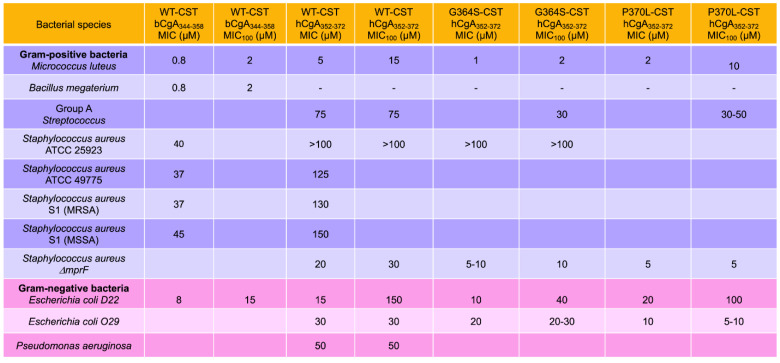
Effects of wild-type (WT)-CST and natural human variants of CST (Gly364Ser and Pro370Leu) on the growth of Gram-positive and Gram-negative bacteria showing minimal inhibitory concentration (MIC) and lethal concentration (MIC_100_) of CST.

The group of Metz-Boutigue first demonstrated the antibacterial activity of CST. Her group used bCST_344–358_ (coining the term cateslytin to describe this antimicrobial effect) to reveal the inhibition of growth of the Gram-positive and Gram-negative bacteria [21]. The minimal inhibitory concentrations (MICs) of CST (bCgA_344–358_, hCgA_352–372_, Gly_364_Ser-CST and Pro_370_Leu-CST) for Gram-positive bacteria (*Micrococcus luteus*, *Bacillus megaterium*, Group A *Streptococcus*, *S. aureus* ATCC 25923, *S. aureus* ATCC 49775, *S. aureus* S1 MRSA, *S. aureus* S1 MSSA, and *S. aureus* DmprF) range from 0.8 µM to >100 µM (Figure 1) [21,24]. The minimal concentration with 100% inhibition (MIC_100_) for Gram-positive bacteria range from 2 µM to >100 µM. The MIC of CST was higher (8 µM to 50 µM) for Gram-negative bacteria (*Escherichia coli* D22, *E. coli* 029, and *Pseudomonas aeruginosa*) compared to Gram-positive bacteria (Figure 1). Likewise, the MIC_100_ of CST was higher (15 µM to 150 µM) for Gram-negative bacteria compared to Gram-positive bacteria (Figure 1). The higher MIC and MIC_100_ values of CST for Gram-negative bacteria are consistent with the presence of extra outer membrane containing lipopolysaccharide (LPS) [25,26]. Beyond the extra-thick cell membrane, Gram-negative bacteria also release exotoxins such as tetanus [27] and cholera toxins [28] that worsen prognosis.

D-bCST_1–15_ was reported to exert more effective antimicrobial effects against various bacterial strains than L-bCST_1–15_ [23]. In addition to its antimicrobial effects, D-bCST_1–15_ was reported to potentiate (additive/synergistic) the antibacterial effects of cefotaxime, amoxicillin, and methicillin [23]. Furthermore, it has been shown that D-bCST_1–15_ neither triggered bacterial resistance nor elicited cytokine release [23]. In addition, D-bCST_1–15_ was reported to be more resistant to degradation by secreted bacterial protease than L-bCST_1–15_ [23]. Thus, it was suggested that D-bCST_1–15_ can be used as a monotherapy or as a combination therapy with currently prescribed antibiotics to counteract various diseases associated with bacterial infection [23].

### 2.2. Composition of Bacterial Membranes

While antibiotics target specific cellular activities (e.g., synthesis of DNA, protein, or cell wall), AMPs target the LPS layer of the cell membrane. Extensive studies have been conducted to learn the composition of the bacterial membrane. The bacterial cytoplasmic membrane consists of zwitterionic phospholipids (phosphatidylcholine, phosphatidylethanolamine, sphingomyelin, etc.) and anionic phospholipids (phosphatidyl serine, phosphatidyl glycerol, etc.), providing them with a negative charge [29,30,31,32]. In contrast, besides the cytoplasmic membrane, Gram-negative bacteria contain an additional strong electronegative LPS-containing thick outer membrane [25,26]. Furthermore, the peptidoglycan layer on the outer side of the cytoplasmic membrane is much thicker in Gram-positive bacteria compared to Gram-negative bacteria (20–80 nm versus ~10 nm) [33,34]. The peptidoglycan layer in Gram-positive bacteria is connected by electronegative wall lipoteichoic acids and anchored on the phospholipid bilayer by electronegative lipoteichoic acids [35]. In contrast, in Gram-negative bacteria, the LPS forms the major lipid component of the outer leaflet of the outer membrane [35].

### 2.3. Secondary Structure of CST Explains the Antibacterial Effects of CST

**Figure 2:**
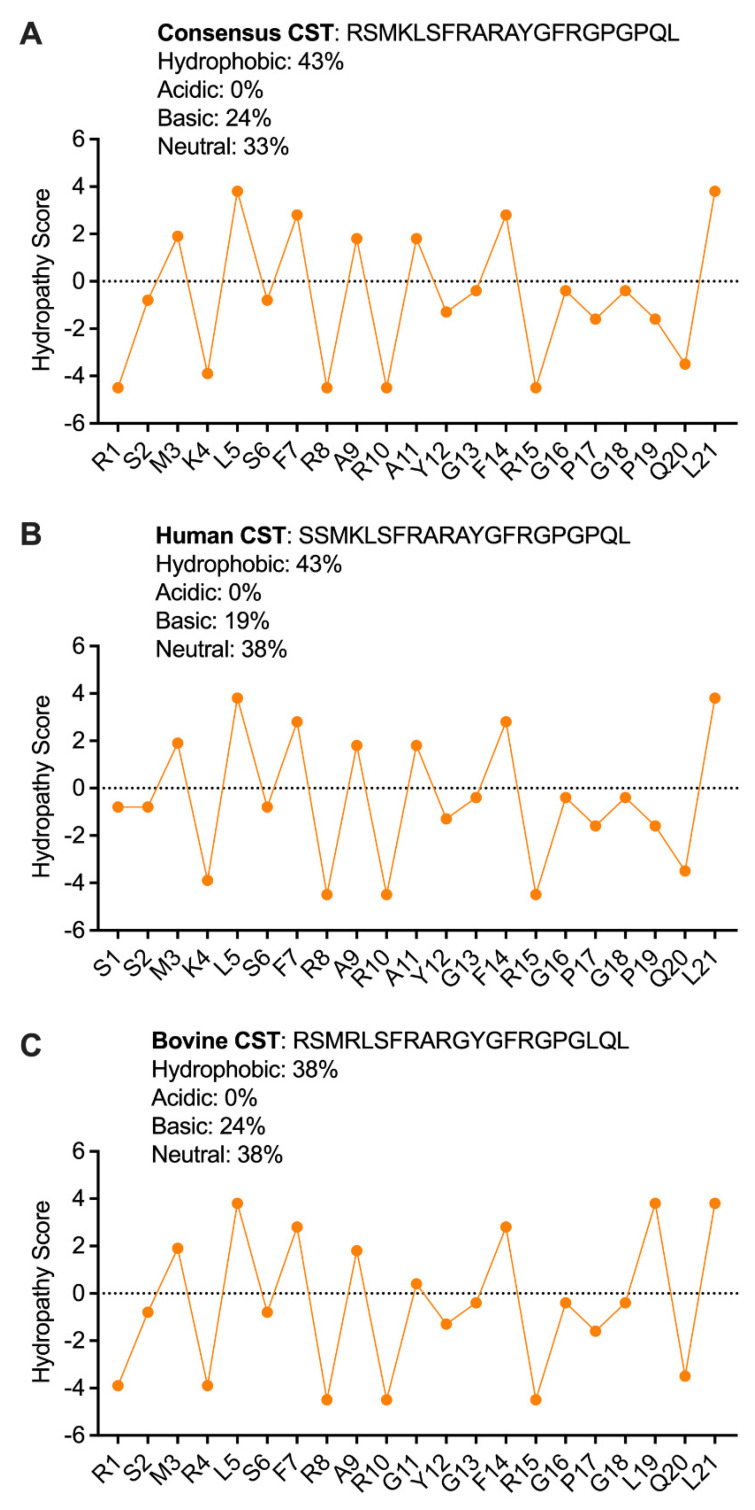
Hydropathy profiles of (**A**) Consensus CST, (**B**) Human CST, and (**C**) Bovine CST. The values are plotted based on the parameters used from Kyte and Doolittle, 1982. The values above zero represents the hydrophobic property of the amino acids that might contribute to the hydrophobic core of the peptide. The values below zero represent the hydrophilic property of the amino acids, which are instrumental in interaction with other protein factors.

Based on their secondary structure, AMPs are generally categorized into four groups: (i) α-helical AMPs, (ii) β-sheet AMPs, (iii) extended AMPs, and cationic loop AMPs [36]. Homology modeling followed by molecular dynamics simulation of bovine CST (bCgA_342–370_) performed in a water shell led to a β-strand-loop-β-strand structure. Molecular dynamics and computer simulations of human CST_1–21_ revealed the following: R_10_, A_11_, and _Y12_ contribute to a 3_10_ helix [37]. In contrast, F_7_, R_8_, A_9_, F_14_, R_15_, G_16_, P_17_, and G_18_ contribute to the antiparallel β-sheet [37]. The mechanism of the antibacterial action of CST_1–21_ could start by interacting with negatively charged moieties such as LPS in the outer membranes of Gram-negative bacteria and lipoteichoic acid in the wall of Gram-positive bacteria. The primary structure of CST reveals that CST contains cationic and hydrophobic residues and adopt a β-sheet secondary structure via intermolecular forces [38]. This folding structure would facilitate CST to fold into an amphiphilic conformation with positively charged (polar) and hydrophobic (nonpolar) faces (Figure 2). The presence of a great number of positively charged residues (5 in bCST and 4 in hCST) will allow CST to interact preferentially with negatively charged bacterial membranes [1,39]. Since the hydrophilic and hydrophobic amino acids of CST are structurally segregated, it will provide solubility of CST in both aqueous and lipid-rich environments, as suggested for other AMPs [40]. In addition, positively charged amino acids in CST formed amphipathic structures, as evidenced by separated hydrophobic and hydrophilic surface domains [39,41] (Figure 2). When the concentration of CST would exceed a certain critical concentration, the cell membrane would form pores, leading to content leakage, cell lysis, and finally death. Since cyclization of peptide has been reported to induce high antimicrobial activity [39,41], it is reasonable to assume that cyclization of CST would markedly improve the antibacterial activity of CST.

Metz-Boutigue’s group has shown that bCgA_344–358_ is unstructured in solution but is converted to an antiparallel β-structure and forms aggregates at the surface of negatively charged bacterial membranes [42]. As for catecholamine secretion [15], arginine residues were found to be crucial for binding to negatively charged lipids [42,43]. They proposed that the phase boundary defects caused by zones of different rigidity and thickness lead to permeability induction and peptide crossing through the bacterial membrane [42]. The fact that CST penetrates through the bacterial wall was shown by measuring the optical density of the released β-galactosidase from ML-35p [24]. Electron microscopical studies of *E. coli* ML-35p confirmed that CST rapidly disrupts the *E. coli* membrane, with visible membrane blebbing compared to untreated cells within 10 min [24].

### 2.4. CST as a Potential Therapy for Bacterial Diseases

AMPs derived from CgA display antimicrobial activities by lytic effects at micromolar range against Gram-positive bacteria, filamentous fungi, and yeasts. Interestingly, CST-derived peptides can kill “superbugs” and more particularly *S. aureus* [44]. Considering the actions of CST on *E. coli*, it could be useful as a therapeutic target for the Gram-negative bacteria that cause many serious infections, including Cholera [45], *E. coli* [46], *Yersinia* [47], *Campylobacter* [48], *Legionella* [49], *Salmonella* [50], *Klebsiella* [51], *Pseudomonas* [52], *Francisella tularensis* [53], *Salmonella typhi* [54], and microbes associated with drug resistance [55,56,57], and CST might be used as a therapeutic target for the above diseases.

## 3. Antifungal and Anti-Yeast Effects of CST

### 3.1. Inhibition of Growth of Fungus and Yeast by CST

**Figure 3:**
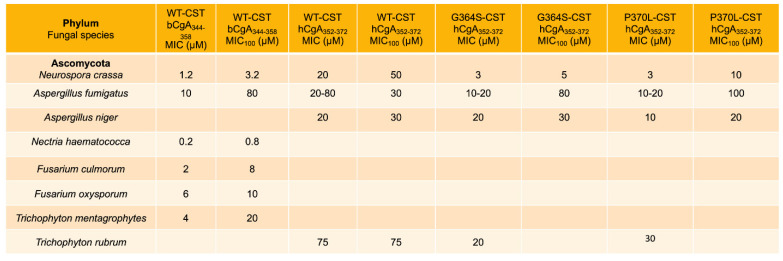
Effects of WT-CST and natural human variants of CST (Gly364Ser and Pro370Leu) on the growth of fungal species showing MIC and MIC_100_ of CST.

**Figure 4:**
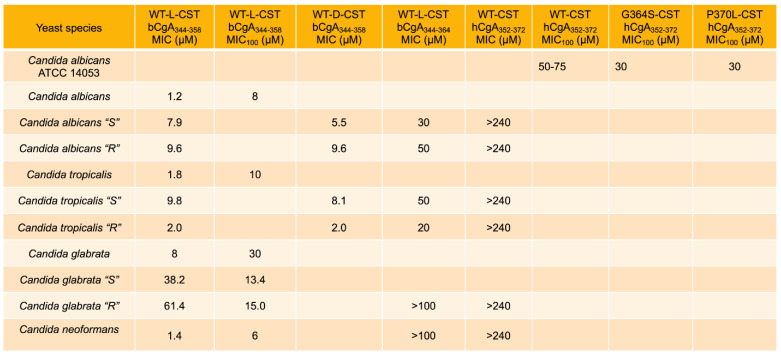
Effects of WT-CST and natural human variants of CST (Gly364Ser and Pro370Leu) on the growth of yeast species showing MIC and MIC_100_ of CST.

Fungal infections are common on the surface of skin, nails, or mucous membranes (superficial or mucocutaneous), underneath the skin (subcutaneous), or in the lungs, brain, or heart (deep infection). Deep fungal infections include Histoplasmosis [58], Coccidioidomycosis (Valley fever) [59], Blastomycosis [60], Aspergillosis [61], Candidal urinary tract infection [62], invasive candidiasis [63,64], *Pneumocystis* pneumonia [65], Mucormycosis [66,67], and Cryptococcosis [68,69]. It is becoming increasingly evident that resistance to antifungal therapy is on the rise [70,71], which calls for the development of alternative therapy for these infections. Host-defense peptides are emerging as new promising candidates to counteract antifungal resistance [72]. To this end, Metz-Boutigue’s group tested the effects of CST on the growth of fungus and yeasts. They found MIC values of CST or its human variants ranging from 0.2 µM to 75 µM against a host of fungal species (*Neurospora crassa*, *Aspergillus fumigatus*, *A. niger*, *Nectria haematococca*, *Fusarium culmorum*, *F. oxysporum*, *Trichophyton mentagrophytes*, and *T. rubrum*) [21,24] (Figure 3). The MIC_100_ values of CST or its human variants against the above fungal species ranged from 0.8 µM to 100 µM [21,24] (Figure 3). CST and its human variants also displayed similar inhibitory effects on the growth of yeasts, with MIC ranging from 1.2 µM to >240 µM (Figure 3) [21,24]. The MIC_100_ of CST and its variants against the above yeasts ranged from 6 µM to 75 µM [21,24] (Figure 4). Similar to the effects of retro-inverso (RI)-CST (Amino-lqpGpGrfGyararfslkmss-carboxyl, with the CST sequence reversed from carboxyl → amino, and chirality was inversed from L → D) on catecholamine secretion [73], D-CST exhibited comparable inhibitory effects on the growth of yeast compared to L-CST with MIC ranged from 2 µM to 9.6 µM [23]. D-CST was also uncovered to be resistant to proteolytic digestion [23,44,74]. Akin to L-CST, D-CST can also be used to develop therapies for drug-resistant microbial infection [75].

### 3.2. Mechanisms Underlying the Antifungal and Antiyeast Activities of CST

**Figure 5:**
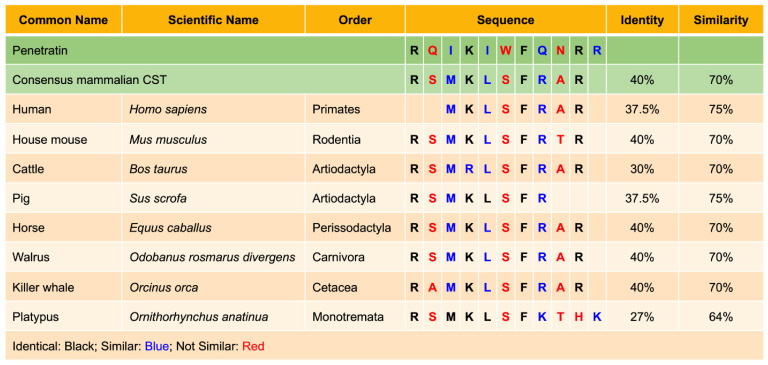
Homology between cell permeable peptide penetratin and CST in seven mammalian orders.

The composition of fungal cell membranes is similar to that of bacteria, comprising zwitterionic and anionic phospholipids. In contrast, the fungal cell wall is composed of chitin, glucan, ergosterol, and mannoprotein, which reside on the surface of the cytoplasmic membrane. Because of the negative charge on the cytoplasmic membrane, CST could exert its anti-fungal activities in a similar way to its antibacterial activity. Metz-Boutigue’s group used confocal laser microscopy to analyze the interaction of the synthetic rhodamine-labeled cateslytin (bCgA_344–358R_) with fungal (*A. fumigatus*) and yeast (*C. albicans*) membranes [21]. Rhodaminated cateslytin (1 µM) was detected in the inner compartment after 2 min of incubation, implicating rapid and efficient penetration through the cell wall [21]. Using time-lapse video microscopy of fungal growth, they have shown that rhodaminated cateslytin blocked the growth and development of nascent fungus [21]. Penetration of rhodaminated cateslytin takes place at both ends of the small fungi (three cells and expressing a slow growth rate) as compared to larger fungi with a higher growth rate where penetration takes place at one end [21]. Sequence homology of the well-known cell-permeable peptide penetratin with CST representing seven mammalian orders (Primates, Rodentia, Artiodactyla, Perissodactyla, Carnivora, Cetacea, and Monotremata) revealed 63.64% to 75% similarity, which should qualify CST as a cell-permeable peptide (Figure 5).

## 4. CST Regulation of Gut Microbiota

### 4.1. Microbiomes in Colonic Mucosa versus Feces

**Figure 6:**
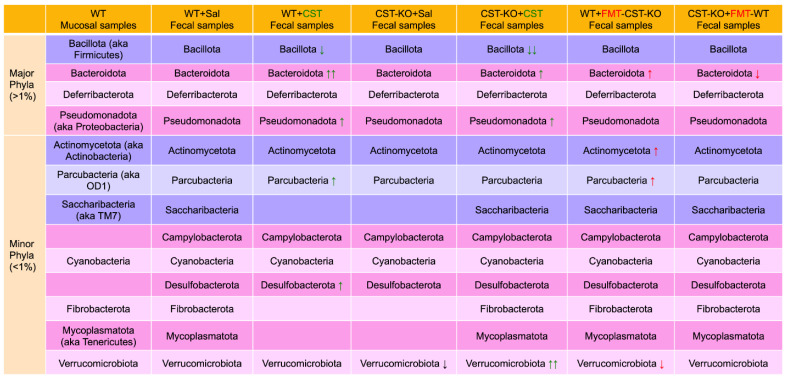
Abundance of bacterial species in mucosal and fecal samples in WT and CST-KO mice in presence or absence of CST as well as after fecal microbial transplant. Green arrows indicate CST effects; red arrows indicate FMT effects.

Recent studies have identified a larger role of gut microbiota in gut-immune homeostasis and in intestinal pathology. The human intestinal microbiota is dominated by five phyla: high-abundant (>80%) (1) Bacillota (aka Firmicutes) and (2) Bacteroidota; less-abundant (3) Actinomycetota (aka Actinobacteria), (4) Pseudomonadota (aka Proteobacteri), and (5) Verrucomicrobiota [76] as compared to four phyla in mice: high-abundant Bacteroidota, Bacillota, Deferribacterota, and Pseudomonadota [77]; and (4) low-abundant Actinomycetota and Verrucomicrobiota compared to humans. In mouse colonic mucosa samples, 19 phyla were identified [78] (Figure 6). Although CST failed to alter bacterial populations in the four high-abundant phyla, it altered colonic mucosa-associated bacterial community composition at lower taxonomic levels, including orders Bacteroidales, Clostridiales, and YS2, and Families Chitinophagaceae, Clostridiaceae, Coriobacteriaceae, Pseudomonadaceae, Rikenellaceae, and Ruminococcaceae [78]. While CST increased the relative abundance of Bacteroidota, it caused a marked decrease in the Bacillota population (Figure 6). *Bacteroides* and *Parabacteroides* species, representing ~25% of the colonic microbiota, transform simple and complex sugars into volatile short-chain fatty acids (SCFAs), such as acetate, butyrate, and propionate [79,80,81], which are absorbed in the colon as a nutrient [82,83]. In addition to colonic nutrients, SCFAs are well established for their roles in accelerating gut transit time via the release of serotonin [84,85]. SCFAs also release glucagon-like peptide 1 from the enteroendocrine L-cells [86,87,88] and improve insulin sensitivity [89,90,91]. *Bacteroides thetaiotaomicron* produces significant amounts of glycosylhydrolases, which prevent obesity [92]. Other *Bacteroides* species are also reported to prevent obesity and increase insulin sensitivity [93,94]. Furthermore, *Bacteroides fragilis* produces zwitterionic polysaccharide, which activates CD4^+^ T cells to produce interleukin 10 (IL-10). IL-10 plays crucial roles in preventing abscess formation and other unchecked inflammatory responses [95,96]. The functional correlation between different CST mutants across species and their respective microbiota has remained elusive.

### 4.2. Microbiomes in CST Knockout (CST-KO) Mice and Inflammation

**Figure 7:**
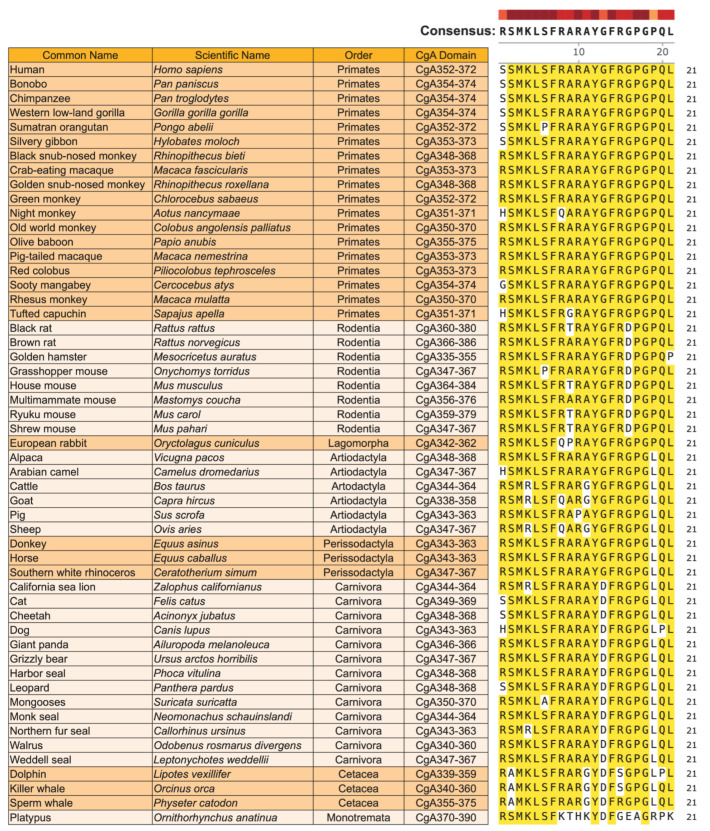
Homology of CST sequence in 53 mammalian species belonging to eight orders. CST sequences were aligned using the MUSCLE method provided by SnapGene software from the following mammalian species: human (Homo sapiens: NM_001275), bonobo (*Pan paniscus*: XP_008956465.1), chimpanzee (*Pan troglodytes*: PNI97600.1), western low-land gorilla (*Gorilla gorilla gorilla*: XM_019009788.2), Sumatran orangutan (*Pongo abelii*: XM_002825045.3), silvery gibbon (*Hylobates moloch*: XP_031990963.1), black snub-nosed monkey (*Rhinopithecus bieti*: XM_017857899.1), crab-eating macaque (*Macaca fascicularis*: XP_045252830.1), golden snub-nosed monkey (*Rhinopithecus roxellana*: XM_010384506.1), green monkey (*Chlorocebus sabaeus*: XM_007987644.2), night monkey (*Aotus nancymaae*: XM_012455409.1), old world monkey (*Colobus angolensis palliates*: XM_011949380.1), olive baboon (*Papio Anubis*: XM_031667888.1), pig-tailed macaque (*Macaca nemestrina*: XM_011717182.1), red colobus (*Piliocolobus tephrosceles*: XM_023205512.3), Sooty mangabey (*Cercocebus atys*: XM_012083744.1), rhesus monkey (*Macaca mulatta*: NM_001278450.1), tufted capuchin (*Sapajus apella*: XM_032287580.1), black rat (*Rattus rattus*: XM_032908276.1), brown rat (*Rattus norvegicus*: XM_032908276.1), golden hamster (*Mesocricetus auratus*: XM_005068386.4), grasshopper mouse (*Onychomys torridus*: XM_036206345.1), house mouse (*Mus musculus*: NM_007693.2), multimammate mouse (*Mastomys coucha*: XM_031357132.1), Ryuku mouse (*Mus caroli*: XM_021179357.1), Shrew mouse (*Mus pahari*: XM_021202342.2), European rabbit (*Oryctolagus cuniculus*: XM_051826432.1), alpaca (*Vicugna pacos*: XP_031534667.1), Arabian camel (*Camelus dromedarius*: XM_031454226.1), cattle (*Bos taurus*: NM_181005.2), goat (*Capra hircus*: XM_018066172.1), pig (*Sus scrofa*: NP_001157477.2), sheep (*Ovis aries*: XP_004018008.3), donkey (*Equus asinus*: XP_014687627.1), horse (*Equus caballus*: NP_001075283.2), southern white rhinoceros (*Ceratotherium simum*: XP_004434274.1), California sea lion (*Zalophus californianus*: XP_027424506.2), cat (*Felis catus*: XP_023111743.1), cheetah (*Acinonyx jubatus*: XP_026922275.1), dog (*Canis lupus*: XP_038528993.1), giant panda (*Ailuropoda melanoleuca*: XP_019660005.1), grizzly bear (*Ursus arctos horribilis*: XP_048075839.1), harbor seal (*Phoca vitulina*: XP_032261715.1), leopard (*Panthera pardus*: XP_019317643.2), mongooses (*Suricata suricatta*: XP_029807749.1), monk seal (*Neomonachus* schauinslandi: XP_021535325.1), Northern fur seal (*Callorhinus ursinus*: XP_025726236.1), walrus (*Odobenus rosmarus divergens*: XP_004394547.1), weddell-seal (*Leptonychotes weddellii*: XP_030873380.1), dolphin (*Lipotes vexillifer*: XP_007454783.1), killer whale (*Orcinus orca*: XP_004262400.1), sperm whale (*Physeter catodon*: XP_023986851.1), and platypus (*Ornithorhynchus anatinua*: XP_039767777.1). Yellow shows an amino acid match between species.

CST knockout (CST-KO) mice were generated in 2018 and are: insulin-resistant on a normal chow diet [97], hyperadrenergic [98], hypertensive [98], and with a leaky gut [99]. The microbiome in CST-KO mice was found to be quite different in composition than its WT littermates [99]. Microbial richness revealed a significant decrease in CST-KO compared to WT mice [100]. (Figure 7). Surprisingly, Verrucomicrobiota population was very low in CST-KO mice, indicating low levels of *Akkermansia* species. Since *A. muciniphila* modulates obesity by regulating metabolism and energy homeostasis to improve insulin sensitivity and glucose homeostasis [101], low Verrucomicrobiota population possibly contributed to the insulin resistance reported for CST-KO mice [97].

### 4.3. Alteration of Diversity and Composition of the Microbiota in the CST-KO after Supplementation with CST

Decreased amplicon sequence variants and abundance-based coverage estimator indices in CST-KO mice were restored after supplementation with CST for 15 days [100]. Akin to richness scores, supplementation of CST-KO mice with CST increased the diversity index as assessed by Shanon’s *H* and inverted Simpson’s index [100]. At the phylum level, CST decreased Bacillota phylum and increased Bacteroidota, Patescibacteria, Desulfobacterota, Verrucomicrobiota, and Proteobacteria in both CST-KO and WT mice [100]. In contrast, CST increased *Alistipes*, *Akkermansia*, and *Roseburia* genera only in CST-KO mice [100].

### 4.4. Restoration of Microbial Dysbiosis in CST-KO Mice after Fecal Microbial Transplant (FMT) from WT Donor Mice

FMT is now established as an effective therapeutic modality in the treatment of the following diseases: (i) antibiotic-refractory recurrent *Clostridium difficile* colitis with a success rate of up to 95% [102,103,104,105], (ii) constipation, (iii) irritable bowel syndrome, and (iv) inflammatory bowel disease [106,107,108]. Therefore, attempts were made recently to assess whether gut microbial population in mice can be reversed by reciprocal FMT. WT mice that received FMT from the CST-KO mice (WT^FMT-CST-KO^) encompassed a reduction of Clostridia and *Akkermansia* [109], which are linked to metabolic disorders and insulin resistance [110,111] and a marked increase in the Proteobacteria population, which are associated with active inflammatory bowel disease (IBD) states [112,113]. Of note, CST-KO mice are insulin-resistant on a normal chow diet [97]. In contrast, insulin-resistant CST-KO mice that received FMT from the WT mice (CST-KO^FMT-WT^) showed an increase in richness, a notable reduction of *Staphylococcus*, and an increase in the butyrate-producing *Intestinimonas* [109] (Figure 6). Butyrate, taken up directly by colonocytes, serves not only as a direct source of energy that contributes directly to a healthy gut, but also acts as a signaling molecule that affects many factors such as satiety, secretion of hormones, and glucose metabolism [114,115,116]. Furthermore, reduced levels of butyrate are strongly associated with IBD and metabolic disorders [117,118]. Butyrate has also been shown to restore gut barrier integrity [119], modulates regulatory T cell function [120,121,122], and regulates certain serine proteases [123,124].

## 5. Catestatin and Innate Immunity

The first indication for the role of CST in innate immunity came from a study in rats where intravenous administration of CST was shown to reduce pressor responses by electrical stimulation [125]. The hypotensive effect of CST was revealed to be mediated at least in part by profuse histamine release (by ~21-fold) and action at the H_1_ receptor [125]. The in vivo studies were later confirmed in peritoneal and pleural mast cells where CST caused dose-dependent release of histamine utilizing signaling pathways established for wasp venom peptide mastoparan and other amphiphilic cationic neuropeptides (the peptidergic pathway) [126]. This pathway is in sharp contrast to the nicotinic-cholinergic pathway used by CST to induce catecholamine secretion from chromaffin cells [5]. Subsequent studies uncover the following: (i) release of immunoreactive CST-containing peptides from human stimulated polymorphonuclear neutrophils [21]; (ii) detection of CST in mouse peritoneal macrophages by Western blots [98]; (iii) detection of CST in human monocytes and monocyte-derived macrophages by Western blots [127]; (iv) blockade of lipopolysaccharide (LPS)-induced increase in expression of tumor necrosis factor alpha [127]; (v) decreased expression of proinflammatory cytokines by CST in plasma and heart [98]; (vi) inhibition of infiltration of macrophages in obese liver [97]; (vii) degranulation of primary mast cells from human peripheral blood [128]; and (viii) low plasma CST in fatal COVID-19 patients [129]. These findings implicate CST as an immunomodulatory peptide. Since receptor-ligand interactions are an essential driver of host-immune response [130], it is important to examine if CST can bind with a receptor on immune cells and regulate their polarization and function in host defense.

## 6. Evolutionary Conservation and Selection Pressure on CST in Mammals

### 6.1. Homology of CST in Mammals

**Figure 8:**
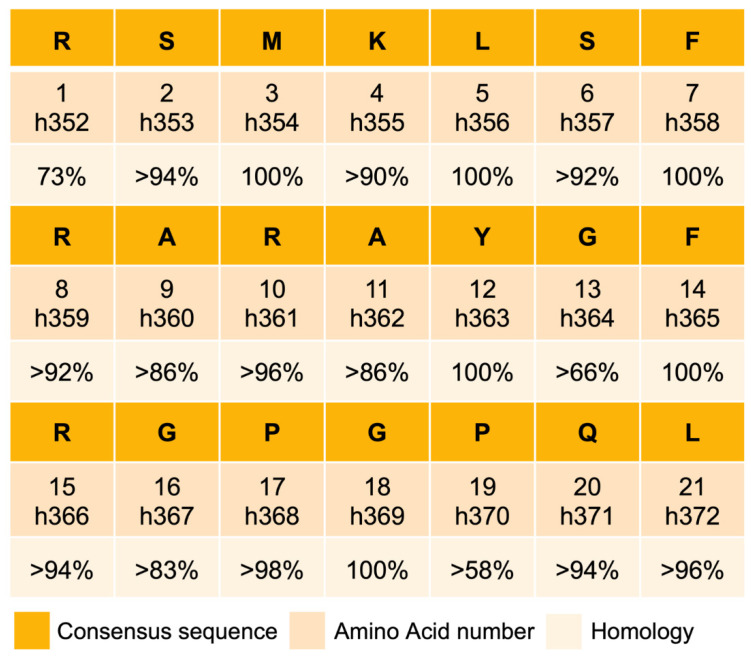
Homology of the individual amino acid in catestatin sequence in 53 mammalian species belonging to seven orders.

Sequence alignment of CST in 53 mammalian species belonging to eight orders revealed >80% homology in 52 species, except in Platypus (lowest in the mammalian phylogenetic tree) where the homology with the primates (highest in the mammalian phylogenetic tree) was >58% (Figure 7), indicating that CST is highly conserved in mammals. The homology of individual amino acids is summarized in Figure 8. Aromatic amino acids such as phenylalanine, tyrosine and tryptophan are reported to exhibit a rigid, planar structure and possess added stability due to the π-electron cloud situated above and below the plane of the aromatic ring [131,132,133]. Therefore, F_7_, Y_12_, F_14_ conserved residues in CST can undergo aromatic–aromatic interactions such as hydrogen bonding coupled with attractive, non-covalent, dipole, and van der Walls interactions, and also pi-stacking of the benzene rings [134,135,136,137]. These interactions, in turn, can stabilize the overall structure of CST, as reported earlier for other proteins [138,139,140,141]. Analysis of the energetics of protein analyses revealed that the packing of non-polar groups in the protein interior is favorable owing to the favorable enthalpy of van der Walls interactions [142]. Therefore, it is reasonable to assume that van der Walls interactions of the aromatic amino acids (F_7_, Y_12_, F_14_) in association with van der Walls interactions of apolar (L_5_, G_18_) amino acids provided a global stability for CST [143,144]. In the course of evolution, with the change in interacting partners across species, we see a significant reduction in the conservation of charged residue. Interestingly, for the maintenance of structural framework, a 100% conservation of hydrophobic amino acid is maintained across the species through the mammalian evolutionary ladder.

### 6.2. Single Nucleotide Polymorphisms (SNPs) in the CST Domain of Mammals

Four non-synonymous SNPs have been identified in CST domain of CgA: Gly_364_Ser (US, Indian, and Japanese populations) [22,145,146], Gly_367_Val (only in Indian populations) [145], Pro_370_Leu (US and Indian populations) [22], and Arg_374_Gln (US populations only) [22]. Pro_370_Leu-CST has the highest potency of inhibiting catecholamine secretion and desensitizing catecholamine secretion, followed by WT-CST and Gly_364_-Ser-CST [22]. As a sharp contrast to catecholamine secretion [22], Gly364Ser was reported to be two-times more effective than Pro370Leu in exerting antibacterial activities [21].

## 7. Conclusions

(i) High conservation of CST in mammals: Alignment of CST sequences from 53 mammalian species belonging to eight orders revealed that CST sequence is highly conserved (>90% in 90% species) in mammals: Five (~24%) amino acids (M_3_, L_5_, F_7_, F_14_, and G_18_) are 100% conserved; nine (~43%) amino acids (S_2_, K_4_, S_6_, R_8_, R_10_, R_15_, P_17_, Q_20_ and L_21_) are 90–96% conserved; and three (~14%) amino acids (A_9_, A_11_, and G_16_) are >80% conserved. The least conserved sequences are G_13_ (>66%) and P_19_ (>58%), where human variants of CST were reported for G_13_ (G_13_S) and P_19_ (P_19_L), indicating that natural selection pressures still exist on those two amino acids [147,148,149,150].

(ii) CST as an immunomodulatory peptide: Existing literature (expression of CST in innate immune cells [21,98,127,151], inhibition of macrophage infiltration in tissues [97,98,99], decreased expression of pro-inflammatory cytokines by CST [97,98], and low plasma CST in fatal COVID-19 patients [129]) implicate CST as an immunomodulatory peptide.

(iii) CST as an antimicrobial peptide: Prominent effects of CST in the low micromolar range on inhibition of growth of Gram-positive and Gram-negative bacteria, fungi, and yeast establish CST as an antimicrobial peptide [21].

(iv) D-bCST_1–15_ as a potential therapy for microbial infection: D-bCST_1–15_ could be used as a monotherapy or as a combination therapy with cefotaxime, amoxicillin, and methicillin against the “superbugs” because it has more effective antibacterial activity compared to L-bCST_1–15_, penetration through the bacterial cell wall, resistance to bacterial proteases, undetectable susceptibility to resistance, and potentiation/synergic action of commonly prescribed antibiotics [23].

(v) CST as a cell permeable peptide: Penetration of CST (pI 12.03–12.48) in bacteria, fungus, yeast, and neutrophils [21,152], coupled with 70–75% homology with cell penetrating peptide Penetratin (pI 12.62), rightfully qualify CST as a cell permeable peptide.

(vi) Gut microbiome-mediated improvement in insulin sensitivity by CST: The increased ratio of Bacilotta to Bacteroidota, together with low levels of Verrucomicrobiota (e.g., *Akkermansia* spp.) in CST-KO mice [100], not only explains insulin resistance in CST-KO mice [97] but also implicates that CST is necessary for the maintenance of insulin sensitivity. A decreased ratio of *Bacilotta* to *Bacteroidota* coupled with increased abundance of *Verrucomicrobiota* after supplementation of CST-KO mice with CST [100] confirm that CST is necessary and sufficient to increase insulin sensitivity by modulating gut microbiota. Decreased population of *Akkermansia* and increased population of Proteobacteria in WT^FMT-CST-KO^ coupled with increased population of butyrate producing *Intestimonas* in CST-KO^FMT-WT^ [109] further substantiates regulation of obesity and insulin resistance by CST [97] via regulation of gut microbial population [100,109].

(vii) Improvement in antimicrobial effect of CST by cyclization: Based on the existing literature [39,41], we propose that cyclization of CST would markedly improve the antibacterial activity of CST.

## Data Availability

Not applicable.
